# Prevalence of loneliness and associations with health behaviours and body mass index in 5835 people living with and beyond cancer: a cross-sectional study

**DOI:** 10.1186/s12889-024-17797-3

**Published:** 2024-02-28

**Authors:** Susan Smith, Phillippa Lally, Andrew Steptoe, Yanaina Chavez-Ugalde, Rebecca J Beeken, Abi Fisher

**Affiliations:** 1 Department of Behavioural Science and Health, University College London, Gower Street, WC1E 6BT London, UK; 2 Department of Psychological Sciences, University of Surrey, GU2 7XH Guildford, Surrey UK; 3 MRC Epidemiology Unit, Institute of Metabolic Science, University of Cambridge School of Clinical Medicine, Cambridge Biomedical Campus, CB2 0QQ Cambridge, Box 285, UK; 4 Leeds Institute of Health Sciences, University of Leeds, LS2 9JT Leeds, UK

**Keywords:** Loneliness, Cancer survivors, Smoking, Exercise, Diet

## Abstract

**Background:**

A cancer diagnosis and its treatment may be an especially isolating experience. Despite evidence that positive health behaviours can improve outcomes for people living with and beyond cancer (LWBC), no studies have examined associations between loneliness and different health behaviours in this population. This study aimed to describe the prevalence of loneliness in a large sample of UK adults LWBC and to explore whether loneliness was associated with multiple health behaviours.

**Methods:**

Participants were adults (aged ≥ 18 years) diagnosed with breast, prostate or colorectal cancer who completed the Health and Lifestyle After Cancer Survey. Loneliness was reported using the UCLA loneliness score, dichotomised into higher (≥ 6) versus lower (< 6) loneliness. Engagement in moderate-to-vigorous physical activity, dietary intake, smoking status, alcohol use, and self-reported height and weight were recorded. Behaviours were coded to reflect meeting or not meeting the World Cancer Research Fund recommendations for people LWBC. Logistic regression analyses explored associations between loneliness and health behaviours. Covariates were age, sex, ethnicity, education, marital status, living situation, cancer type, spread and treatment, time since treatment, time since diagnosis and number of comorbid conditions. Multiple imputation was used to account for missing data.

**Results:**

5835 participants, mean age 67.4 (standard deviation = 11.8) years, completed the survey. 56% were female (*n* = 3266) and 44% (*n* = 2553) male, and 48% (*n* = 2786) were living with or beyond breast cancer, 32% (*n* = 1839) prostate, and 21% (*n* = 1210) colorectal. Of 5485 who completed the loneliness scale, 81% (*n* = 4423) of participants reported lower and 19% (*n* = 1035) higher loneliness. After adjustment for confounders, those reporting higher levels of loneliness had lower odds of meeting the WCRF recommendations for moderate-to-vigorous physical activity (Odds Ratio [OR] 0.78, 95% Confidence Internal [CI], 0.67, 0.97, *p* =.028), fruit and vegetable intake (OR 0.81, CI 0.67, 1.00, *p* =.046), and smoking (OR 0.62, 0.46, 0.84, *p* =.003). No association was observed between loneliness and the other dietary behaviours, alcohol, or body mass index.

**Conclusions:**

Loneliness is relatively common in people LWBC and may represent an unmet need. People LWBC who experience higher levels of loneliness may need additional support to improve their health behaviours.

**Supplementary Information:**

The online version contains supplementary material available at 10.1186/s12889-024-17797-3.

## Background

There are over 3 million people living with and beyond a cancer diagnosis (LWBC) in the United Kingdom (UK) [1]. Assuming trends continue, this will increase by 1 million per decade to 2040 [2]. Similar increases have been observed in other developed countries [3]. As cancer survival rates continue to improve, it is crucial to ensure that people LWBC are supported to achieve the best quality of life, a key aim of cancer strategies [4, 5]. Loneliness (the subjective negative experience of social isolation [6, 7]) is an established risk factor for poorer health and higher mortality in the general population [8–11], and there is some indication that loneliness may result in worse outcomes after a cancer diagnosis [12].

In a meta-analysis of 87 prospective cohorts of cancer patients, factors associated with lower loneliness, such as greater social network size, higher perceived social support, and being married, were associated with 12–25% lower mortality, although a measure of loneliness itself was not included [13]. Furthermore, a synthesis of 20 qualitative studies identified sources of loneliness specifically related to a cancer diagnosis and treatments, including feeling alone in the experience, others’ avoidance of discussion about cancer, lack of understanding/misperceptions of cancer, lack of recognition of the impact of the side effects of treatment, and unmet needs in the healthcare system [14]. For instance, Rosedale described how women diagnosed with breast cancer reported that their healthy peers had difficulty grasping the magnitude of the challenges they faced. They described feeling left behind as others continued with their lives, resulting in feelings of loneliness, disconnectedness, and distress [15]. A meta-analysis of 15 observational studies describing the prevalence of loneliness after a cancer diagnosis found moderate prevalence and associations with a number of demographic and clinical factors [16]. However, the samples within the included studies were small (13/15 included ≤ 200 participants), and larger studies exploring loneliness after acute cancer treatment phases are needed.

Loneliness may impact health outcomes via both direct biological mechanisms and behavioural mechanisms [17, 18]. For example, loneliness may have a negative impact on health behaviours in people LWBC and an ever-growing body of evidence suggests that healthier behaviours lead to better outcomes after a cancer diagnosis [19–22]. This has led a number of governing bodies, such as the World Cancer Research Fund and American Institute for Cancer Research (WCRF/AICR), to issue health behaviour guidance for those LWBC [23, 24]. There is already evidence to suggest that some health behaviours worsen following a diagnosis of cancer and its treatments (e.g., decline in physical activity [25, 26]), so negative effects could be exacerbated in lonelier people. However, data exploring associations between loneliness and health behaviours are mainly from the general population. A systematic review of 37 studies exploring the association between loneliness and physical activity, including healthy older adults and adolescents, found negative associations between physical activity and loneliness in 12 cross-sectional studies and one longitudinal study [27]. Although relationships are likely to be bidirectional, four prospective studies have found that people with higher levels of loneliness at baseline were less likely to be physically active at follow-up [28–31]. While Newall and colleagues reported that loneliness predicted perceived engagement in physical activity in a sample of 228 older adults, they also found that this relationship was moderated by happiness, suggesting that happiness may buffer against the negative effect of loneliness in this age group [29]. In a study of older adults taking part in the English Longitudinal Study of Ageing (ELSA), Kobayashi and Steptoe investigated the associations between social isolation and loneliness at baseline and engagement in health behaviours over 10 years [32]. After dichotomisation of health behaviours into meeting or failing to meet the public health recommendations, they observed that higher loneliness was associated with lower odds of meeting the recommendations for physical activity, smoking and body mass index (BMI), although the association between loneliness and health behaviours was lost after adjustment for sociodemographic variables [32, 33]. Similarly, where Schrempf and colleagues examined how objective physical activity differs according to levels of social isolation and loneliness in ELSA, a negative association was observed between total physical activity counts and loneliness, although this was attenuated after adjusting for covariates [33]. Data exploring associations between loneliness and physical activity in people LWBC are lacking. In older breast cancer survivors, one prospective study found an inverse association between loneliness and physical activity over a five-year follow-up period [34], while another cross-sectional study found that lower social support and living alone was associated with lower levels of physical activity [35].

In addition to physical activity, there is also some evidence that loneliness can also impact health via smoking and poor dietary intake. A systematic review of studies on loneliness and smoking reported that over half of the 23 included studies observed an association between smoking and loneliness, and their results consistently demonstrated that lonely people were more likely to be smokers [36]. This association is likely to be bidirectional whereby smokers are also more likely to be lonely [37]. Despite qualitative data suggesting that experiencing loneliness may negatively impact eating habits in older adults [38, 39], quantitative data exploring associations between loneliness and intake of different dietary components are lacking. Lastly, loneliness may impact body mass index (BMI) in people LWBC, as evidence from the general population suggests a complex association between loneliness and obesity due to factors such as weight-related stigma [40, 41]. While there is conflicting evidence suggesting a BMI in the overweight range (25-29.9) may be associated with improved survival across some cancer types, explanations for this paradox have still yet to be established [42, 43]. Acknowledging that this evidence is still inadequate to make specific recommendations with confidence, the WCRF recommends that people LWBC follow the guidelines published for healthy populations in maintaining a BMI in the healthy range (18.5–24.9), recognising that this is unlikely to be harmful to people LWBC who have completed treatment [42].

While the link between loneliness and health behaviours in this population remains underexplored, behaviour change theories such as social cognitive theory (SCT [44]) and the capability, opportunity, and motivation model of behaviour (COM-B [45]) can complement each other in providing us with possible explanations for this relationship. In line with SCT, it is plausible that individuals with LWBC experiencing loneliness may experience low self-efficacy attributed to feeling a loss of control over their health and how others react [14], and this lack of confidence can hinder their ability to engage in behaviours such as physical activity [46]. This is also emphasised by the COM-B model where reduced capability can erode the individual’s confidence and belief in their ability to perform positive health behaviours. Furthermore, the opportunity component of the COM-B model asserts that the environment must make the execution of the behaviour possible [45]. As lonelier people LWBC may have fewer supportive networks to encourage and facilitate participation in health-promoting behaviours, their opportunity to perform these behaviours can be limited [15].

The aims of the current study were to (i) describe the prevalence of loneliness in a large sample of LWBC in the UK and (ii) explore associations between loneliness and health behaviours while adjusting for sociodemographic and clinical factors.

## Methods

### Design and participants

The ‘Health and Lifestyle after Cancer’ survey was mailed to patients who had received a diagnosis of breast, prostate, or colorectal cancer between 2012 and 2015 at 10 participating National Health Service (NHS) hospital sites in London and Essex (United Kingdom). Dates of diagnosis for survey administration were chosen because the survey was also used to identify initial interest in the Advancing Survival after Cancer Outcomes Trial (ASCOT) (which included only patients who had completely primary curative treatment) [47]. Patients were identified by hospital staff and although primarily included patients diagnosed between 2012 and 2015, the final sample for the analysis included patients diagnosed with breast, prostate, or colorectal cancer outside of these dates (range: 1994–2017). As some participants had received a subsequent cancer diagnosis, the date of their most recent breast, prostate, or colorectal cancer diagnosis was reported and used in the analysis. This ranged between 2001 and 2017 in the final sample (mean time in months = 35.5, standard deviation (SD) = 13.6). Survey packs were sent between February 2015 and November 2017 and included a letter of invitation signed by the hospital consultant, a paper version of the survey, and a link to an online version. Participants completed the survey via their preferred method and returned it directly to the research team. Returned questionnaires were accepted until 4th January 2018. No data were collected on non-responders.

### Inclusion and exclusion

Inclusion criteria were adults (≥ 18 years) who had received a diagnosis of breast, prostate, or colorectal cancer in one of the participating hospitals. Survey exclusion criteria were intentionally minimal to make administration at hospital sites as low-burden as possible. Patients were only excluded if it was identified that they were deceased or if hospital staff deemed it inappropriate to send the patient a questionnaire for any other reason (e.g., they had previously requested not to be approached about participation in research).

### Ethical approval

This study received ethical approval from the NHS National Research Ethics Committee– South Central Oxford B (reference 14/SC/1369). The initial page of the survey stated that completion and return of the questionnaire meant giving consent for the anonymous collected data to be used in research on lifestyle in people diagnosed with cancer.

### Demographic and clinical characteristics

Age was recorded as a continuous variable (in years). Sex (male/female), highest level of education (no formal qualifications/General Certificate of Secondary Education,Vocational or equivalent/A-levelor equivalent/Bachelor’s Degree and above), marital status (married/divorced or separated or widowed or single), and living arrangements (alone/with others) were recorded. Ethnicity information was collected by 15 possible responses to the question “Which of these best describes your ethnic group?”. For cancer-related questions, participants were asked to answer in relation to their most recent cancer diagnosis, given the questionnaire was sent based on their diagnosis of breast, prostate, or colorectal cancer in 2012 or 2013. Participants were asked to report their cancer stage (1/2/3/4/don’t know), but because a high proportion (43%) didn’t know, ‘has your cancer spread’ (yes/no) was used as a proxy (after confirmation from an oncologist that this was acceptable). Cancer treatments (no treatment or active surveillance/surgery/surgery plus one other/other combination of treatment) and time since main treatment completed (< 1 year/1–5 years/on active surveillance) were reported. Time since diagnosis was calculated as the time between their most recent cancer diagnosis date and the date when the questionnaire was received back by the research team (in months). Participants self-reported their comorbidities from a pre-defined list including 15conditions (osteoporosis/diabetes/asthma/emotional or psychiatric illness/stroke/Parkinsons disease/Alzheimer’s disease or dementia/lung disease/arthritis/angina/heart attack/heart murmur/irregular heart rhythm/any other heart trouble/another cancer) and could report any ‘other’ comorbidities that were not present on the list. Total comorbidities were calculated by adding these together and where participants did not report having any of these conditions, this was interpreted as having no comorbidities. Self-reporting of comorbid conditions has shown a high level of accuracy when compared with medical records in people LWBC [48, 49].

### Exposure: loneliness

Loneliness was assessed using the 3-item short form of the Revised UCLA Loneliness Scale [50]. The UCLA scale has been the most commonly used to assess loneliness after cancer [16], and items in the short form are ‘How often do you feel you lack companionship?’, ‘How often do you feel left out?’ and ‘How often do you feel isolated from others?’ with response options: ‘1 = Hardly ever or never’, ‘2 = Some of the time’, and ‘3 = Often’. Scores for each item are summed to create a total loneliness score that can range from 3 to 9. Total scores were not normally distributed, so they were dichotomised to represent higher (≥ 6) and lower loneliness (< 6). This approach was taken in previous research in the English Longitudinal Study of Ageing [32], allowing for descriptive comparison of the prevalence of loneliness with a representative sample of older adults in the general population.

### Outcomes: health behaviours and BMI

The guidelines for each behaviour for people LWBC were taken from the WCRF/AICR [4] and national UK guidelines, and these were coded as meeting vs. not meeting guidelines.

### Physical activity

Weekly minutes of moderate to vigorous physical activity (MVPA) were assessed using the Godin Leisure Time Exercise Questionnaire (GLTEQ). The GLTEQ is the most commonly used self-report measure of activity in oncology and compares favourably to objective measures [51–53]. The questionnaire was modified to include a question about duration of activity to allow calculation of minutes of MVPA, which is a very common approach in oncology research [52]. MVPA was dichotomised as meeting (≥ 150 min/week) or not meeting (< 150 min/week) recommendations.

### Diet

The validated Dietary Instrument for Nutrition Education Food Frequency Questionnaire (DINE FFQ) [54] was used to assess dietary intake including fibre and fat intake, with some food items updated to reflect those currently available and items amended to include red and processed meat estimation [47]. This measure has been previously validated in the general population [54] and was chosen after a review of validated food frequency questionnaires and a review of how diet has previously been assessed in people LWBC [47]. To estimate free sugar, the survey asked about consumption of sugary drinks and fruit juices [55] and included a custom-made question asking participants to write their total number of teaspoons of added sugar per day. Two items were included to measure the number of daily portions of fruit and vegetables [56]. This measure has demonstrated sufficient validity when compared against blood chemistry [56, 57]. The cut-off scores for meeting the WCRF recommendations were as follows: fruit and vegetables (≥ 5 portions/day), fibre (≥ 30 g Association of Official Analytical Collaboration fibre [58]/day), free sugar (< 5% calories from free sugars/day), fat (< 33% total energy), red meat (< 500 g/week), and processed meat (none). The scoring system implemented for operationalising meeting or not meeting the recommendations has been described previously [59].

### Alcohol & smoking

Smoking status was collected with a single item from the Health Survey for England to categorise participants as current smokers (not meeting) or non-smokers (meeting) [60]. Alcohol consumption was self-reported with two items (How often do you have a drink containing alcohol?; How many units of alcohol do you drink on a typical day when you are drinking?), adapted from The Alcohol Use Disorders Identifcation Test consumption questions (AUDIT-C [61]) which were calculated into an estimate of the average number of units consumed per week. The use of the AUDIT in its original and reduced form has been validated across different contexts and cultures [62]. Meeting or not meeting this recommendation was operationalised based on the national UK guidelines for alcohol consumption of not exceeding more than 14 units of alcohol per week [63].

### Body mass index

BMI was calculated from participants’ self-reported height and weight. Participants were classified into the following categories: underweight, healthy, overweight, or obese [64]. Meeting the WCRF recommendation for BMI was operationalised as being classified into the healthy weight category (18.5–24.9 kg/m^2^).

### Covariates

Demographic and clinical variables that have previously been associated with loneliness and/or health behaviours were included as covariates [16, 65–71]. This included age, sex, level of education, marital status, and living arrangements. Ethnicity was dichotomised (white/any other ethnicity) due to a very high proportion reporting being white (90%). ‘Has your cancer spread’ was used as a proxy to determine cancer stage. Type of cancer, type of treatment, time since diagnosis, time since completion of main treatment, and number of reported comorbidities were also included.

### Analyses

IBM Statistical Package for the Social Sciences (SPSS) version 26 was used [72]. Descriptive statistics were used to present participant characteristics and to describe the proportion of people reporting higher versus lower loneliness (Aim i).

Missing value analysis found that 5.5% of 518,348 values were missing and that 27.8% of 5,835 cases had at least 1 piece of missing data. Multiple imputation with 20 iterations was conducted to account for missing data [73].

To examine whether loneliness was associated with health behaviours (Aim ii), a series of binary logistic regressions were run with loneliness as the exposure, each WCRF health behaviour recommendation as the outcome and adjusting for all covariates. To avoid the ‘Table 2 fallacy’ [74], only the odds ratio (OR) and 95% confidence interval (CI) for the associations between exposure and outcomes are presented here. Regression analyses were repeated in the non-imputed original data to explore whether findings were similar.

## Results

A total of 13,645 surveys were sent, and 5835 were returned (43%response rate). No data were collected on non-responders.

### Descriptive statistics

Demographic and clinical characteristics are presented in Table 1. Participants mean age was 67 years (SD = 12, range 26–97). 44% (*n* = 2553) were male and 56% (*n* = 3266) were female. 48% (*n* = 2786) were living with or beyond breast cancer, 32% (*n* = 1839) prostate and 21% (*n* = 1210) colorectal.

**Table 1:** Participant characteristics

Variable	Respondents (*N* = 5835)
**Sex n(%)**	
Male	2553 (43.8)
Female	3266 (56.0)
Missing	16 (0.3)
**Age in years**	
Mean (SD^a^)	67.4 (11.8)
Missing n(%)	36 (0.6)
**Highest education n(%)**	
None	1709 (29.3)
General Certificate of Secondary Education/Vocational	1613 (27.6)
A-Level	584 (10.0)
Degree or above	1379 (23.6)
Missing	550 (9.4)
**Marital status n(%)**	
Married	4037 (69.2)
Divorced/Separated/Widowed/Single	1781 (30.5)
Missing	17 (0.3)
**Living Arrangements n(%)**	
Alone	1268 (21.7)
With others	4526 (77.6)
Missing	41 (0.7)
**Ethnicity– dichotomised n(%)**	
White	5249 (90.0)
Any other ethnicity	554 (9.5)
Missing	32 (0.5)
**Months since most recent cancer diagnosis**	
Mean (SD^a^)	35.5 (13.6)
**Cancer type n(%)**	
Breast	2786 (47.7)
Prostate	1839 (31.5)
Colorectal	1210 (20.7)
Missing	0
**Cancer spread n(%)**	
Yes	558 (9.6)
No	4498 (77.1)
Don’t know/Missing	373 (6.4)
Missing	406 (7.0)
**Treatment n(%)**	
No treatment/active surveillance only	296 (5.1)
Surgery only	1081 (18.5)
Surgery and at least one other treatment	2967 (50.8)
Any other combination of treatment	1411 (24.2)
Missing	80 (1.4)
**Time since completion of main treatment** ^b^ **n(%)**	
< 1 year	985 (16.9)
1–5 years	4122 (70.6)
On active surveillance	525 (9.0)
Missing/Don’t know	203 (3.5)
**Total comorbidities** ^**c**^	
Mean (SD^a^)	1.3 (1.3)
Missing	0

^a^SD=standard deviation.^b^Time since completion of initial treatment for their cancer that was diagnosed between 2012 and 2015. ^c^Not selecting any condition was interpreted as having no comorbidities, resulting in no missing data for this variable.

Descriptive data for loneliness and health behaviours are presented in Table 2. 76% (*n* = 4423) participants reported lower loneliness, and 18% (*n* = 1035) reported higher loneliness. 377 participants (7%) had missing data. Of the 5485 participants who completed the loneliness scale, 81% (*n* = 4423) reported lower and 19% (1035) reported higher loneliness. A descriptive comparison of those with complete data versus imputed data is presented in Additional file 1.

**Table 2:** Descriptive statistics of loneliness and meeting the recommendations for health behaviours^a^

Variable	Respondents (*N* = 5835)
**Loneliness n(%)**	
Higher	1035 (17.7)
Lower	4423 (75.8)
Missing	377 (6.5)
**MVPA**^b^ **recommendations n(%)**	
Not meeting	3359 (57.6)
Meeting	1790 (30.7)
Missing	686 (11.8)
**Fibre recommendations n(%)**	
Not meeting	3914 (67.1)
Meeting	667 (11.4)
Missing	1254 (21.5)
**Fruit and Vegetable recommendations n(%)**	
Not meeting	4006 (68.7)
Meeting	1659 (28.4)
Missing	170 (2.9)
**Fat recommendations n(%)**	
Not meeting	1769 (30.3)
Meeting	2300 (39.4)
Missing	1766 (30.3)
**Sugar recommendations n(%)**	
Not meeting	2663 (45.6)
Meeting	2694 (46.2)
Missing	478 (8.2)
**Red meat recommendations n(%)**	
Not meeting	139 (2.4)
Meeting	5035 (86.3)
Missing	661 (11.3)
**Processed meat recommendations n(%)**	
Not meeting	2861 (49.0)
Meeting	2640 (45.2)
Missing	334 (5.7)
**Alcohol recommendations n(%)**	
Not meeting	714 (12.2)
Meeting	4848 (83.1)
Missing	273 (4.7)
**Smoking recommendations n(%)**	
Not meeting (i.e., current smoker)	347 (5.9)
Meeting (i.e., current nonsmoker)	5445 (93.3)
Missing	43 (0.7)
**BMI**^c^ **recommendations n(%)**	
Not meeting	3521 (60.3)
Meeting	1978 (33.9)
Missing	336 (5.8)

^a^Meeting and not meeting the recommendations is based on the World Cancer Research Fund recommendations (42). ^b^MVPA=moderate-to-vigorous physical activity. ^c^BMI=body mass index.

### Regression results

Pooled (20 iterations) associations between loneliness and health behaviours are presented in Table 3. After adjustment for covariates, those reporting higher levels of loneliness had lower odds of meeting the WCRF recommendations for MVPA (OR 0.78, 95% CI 0.67, 0.97), fruit and vegetables (OR 0.81, CI 0.67, 1.00), and smoking (OR 0.62, 0.46, 0.84), but not other dietary recommendations (fibre, red or processed meat, sugar, fat– OR and CIs presented in Table 3), alcohol (OR 0.89, CI 0.67, 1.19) or BMI (0.79, CI 0.46, 1.16).

**Table 3:** Pooled (20 iterations) associations between loneliness and health behaviours in 5835 people LWBC^a^

World Cancer Research Fund recommendations (not meeting = 0, meeting = 1)	OR^b^	95% CI^c^		*p*
Moderate-to-vigorous physical activity	0.78	0.67	0.97	0.028*
Fibre	1.07	0.79	1.44	0.663
Fruit and vegetables	0.81	0.67	1.00^d^	0.046*
Red Meat	0.72	0.45	1.15	0.163
Processed Meat	1.00	0.83	1.19	0.954
Sugar	0.84	0.70	1.00	0.068
Fat	0.87	0.69	1.04	0.249
Alcohol	0.89	0.67	1.19	0.424
Smoking	0.62	0.46	0.84	0.003*
Body mass index	0.79	0.46	1.16	0.790

^a^Reference category: lower loneliness (< 6 on UCLA loneliness scale). Models adjusted for covariates– age, sex, ethnicity, education, marital status, cancer type, treatments, time since treatment, time since diagnosis, number of comorbidities, cancer spread; ^b^OR=odds ratio; ^c^CI=Confidence interval. ^d^0.996 rounded up *indicates statistical significance at 0.05 alpha level.

### Analysis with the original data

Logistic regression analyses with the complete case data showed similar associations to the imputed data (see Additional file 1).

## Discussion

In this sample of 5835 people LWBC, 76% reported experiencing lower levels of loneliness, while 18% reported higher loneliness. Individuals who reported higher levels of loneliness were less likely to meet the WCRF recommendations for MVPA, fruit and vegetable intake, and smoking (i.e. they are less likely to do the recommended levels of activity (150 min a week), they are less likely to eat 5 portions of fruit and vegetables a day and they are more likely to be smokers). No association was observed between loneliness and meeting the recommendations for fibre, red or processed meat, fat, sugar, or alcohol and BMI.

The results of this study demonstrate slightly lower levels of loneliness in people diagnosed with breast, prostate, or colorectal cancer than previous studies conducted in people LWBC (22-36%) [75, 76]. This discrepancy might be attributed to the dichotomisation of UCLA scores in this study, resulting in an arbitrary definition of being lonely or not lonely. It therefore is not directly comparable to other studies using different scales and thresholds, for example, De Boer and colleague’s study in breast cancer patients used the De Jong Gierveld Scale [66], where a score above three was interpreted to mean experiencing loneliness [76]. However, where the same scale and threshold for loneliness was applied in the general population in the UK, the prevalence of loneliness in people LWBC was similar (16%; [32]). Differences in the prevalence of loneliness may also be attributed to the timing of the study, with the majority (70%) of participants being 1 to 5 years post finishing their primary treatment. Loneliness levels are likely to fluctuate considerably within this period, with loneliness scores in studies conducted during or just after initial treatment for cancer being significantly lower than loneliness scores in studies conducted in participants more than a year after diagnosis [16]. This finding is also supported by qualitative research [15, 77] and may be attributed to the plausible increase in social support in the period immediately after diagnosis and initial treatment [75].

Although the effect size observed was small, the results of this study converge with previous research reporting a decreased likelihood of engaging in physical activity in people who report higher levels of loneliness [18] and extend sparse findings of an association between loneliness and physical activity in people LWBC [34, 35]. Lemij and colleagues' study in older women (aged 70+) diagnosed with breast cancer reported that increasing loneliness was associated with lower levels of physical activity over time [46]. Although the current study used a different approach to dichotomise physical activity and loneliness, the results still support this inverse association in a younger, larger sample of adults diagnosed with breast, prostate, or colorectal cancer. This is important because there is strong evidence of improved outcomes for people LWBC who engage in higher levels of physical activity, including increased chances of survival and improvements in psychosocial outcomes [20, 78, 79]. Although this study was cross-sectional, it has identified a need for the consideration of psychosocial variables such as loneliness in efforts to increase physical activity in this population. While physical activity interventions in oncology often include a social component and encourage social support [80, 81], a more targeted approach may be needed to address the subjective experience of loneliness. Given the distinction between social isolation and loneliness, efforts to increase social contact and social support may not necessarily address aspects of loneliness, including sense of belonging and feeling cared for by others [82]. Supporting this, Dowd and colleagues reported that an intervention where physical activity was framed as being beneficial for both health and social skills led to increased engagement in physical activity while simultaneously improving loneliness in university students [83]. The results of the current study provide scope for investigating whether this type of interventional approach may also show promise in people LWBC.

The WCRF recommends that people LWBC follow a healthy diet by adhering to the published dietary recommendations for primary cancer prevention [23]. To the authors’ knowledge, this is the first study to quantitatively explore loneliness and specific dietary component intake in people LWBC and highlights that experiencing loneliness may contribute to making poorer dietary choices. Given the sparse evidence base, more research is needed to understand the potential reasons for a lack of association between loneliness and meeting the recommendations for red and processed meat, fat, fibre, and alcohol. In the current study, this may be due to the limited variation in the sample, with a high percentage of participants meeting some of the recommendations (e.g., red meat and alcohol). Loneliness was associated with fruit and vegetable consumption in this sample, where participants who reported experiencing higher levels of loneliness were less likely to meet this dietary recommendation. However, it is important to note that this effect size was small, and the confidence intervals suggest that the impact of loneliness on intake of fruit and vegetable may be relatively weak. Richard and colleagues’ also found that lonelier men and women in a Swiss national survey had worse adherence to the recommendations for fruit and vegetables, although their study measured adherence using a subjective question on whether they perceive that they meet the recommendations, rather than being calculated from a food frequency questionnaire [84]. The observed association in this study is small, but diverges from Kobayashi and colleagues’ finding of no relationship between meeting the fruit and vegetable recommendations and loneliness in the UK general population [32], and instead suggests that people diagnosed with cancer may be particularly susceptible to the negative impact of loneliness on their eating behaviours. More research is needed to investigate the potential mechanisms that underlie this relationship, to ultimately inform targeted intervention design in this population.

Our finding that loneliness was associated with smoking in people LWBC is in line with findings from a systematic review of loneliness and smoking, whereby lonely people were more likely to be smokers [36]. More recently, this association has also been found in a longitudinal analysis of the UK Biobank data, where Eloivaino and colleagues reported that smoking, alcohol, and physical activity accounted for 32-54% of the excess risk of mortality associated with loneliness in the general population [85]. Furthermore, previous research indicates a bidirectional association, where smokers are also more likely to experience higher loneliness over time [37]. In the current study, the possibility of reverse causality in associations between loneliness and health behaviours cannot be ruled out. Specifically, it is possible that loneliness may be influenced by engagement in certain health behaviours. However, with the available cross-sectional data, it is challenging to determine the temporal ordering to provide a more comprehensive understanding of causality in the observed associations. In any case, bidirectional evidence suggests a vicious cycle wherein loneliness and unfavourable health behaviours reinforce one another [27, 36, 37]. Further research, particularly longitudinal studies, is needed to unravel the complexities of the relationship between loneliness and health behaviours and to understand the directionality and underlying mechanisms involved. Using a longitudinal design would also help identify if there are critical periods where loneliness may be particularly influential on health behaviours. This is particularly important for this population as people LWBC move between the phases of diagnosis, treatment, and survivorship and each of these phases present unique demands and challenges to the individual [86].

The mechanisms linking loneliness and health behaviours remain relatively unexplored, but it is important to acknowledge the potential mediating role of depression. Loneliness and depression are considered to be reciprocally associated [9, 87], therefore, it may be that depression lies on a causal pathway where negative affect resulting from loneliness impacts health behaviours [32]. As the data were cross-sectional, no mediation analysis was conducted and accordingly, depression was not included in the model as it was deemed inappropriate to control for potential mediators [88, 89]. Additionally, social interactions and norms can shape health behaviours and lonelier people may not be as exposed to others engaging in practices such as healthy eating and exercise due to reduced social engagement [90, 91]. In line with the recognition of the social environment in SCT [44] and the opportunity component of the COM-B model [45], studies in both the general population and people LWBC have demonstrated that witnessing health-promoting behaviours is associated with increased likelihood of engaging in these behaviours [92–95]. Lastly, as demonstrated in Newall and colleagues’ study in the general population, happiness may moderate this association whereby higher levels of happiness may weaken the association between loneliness and unhealthy behaviours [29]. This may be attributed to the broadening influence of positive emotions, which can counteract the negative impact of loneliness [29, 96]. Future research should employ qualitative methods to uncover the nuances in these associations and to explore potential mechanisms. For example, qualitative interviews with a sample of people with type 2 diabetes revealed reduced motivation due to loneliness and reduced social contact inhibited engagement in physical activity during the COVID-19 pandemic [97].

The results of this study can inform policy and practice by directing support toward the creation of environments that promote social connections and reduce loneliness in this population. Addressing loneliness is a key part of public health agendas with strategies such as social prescribing being implemented into the NHS Long Term Plan [98]. This type of approach aims to combat loneliness by enabling individuals to co-develop solutions to help them cope with their health, while developing connections with communities and building social engagement [99, 100]. The impact of this approach on reducing the negative effect of loneliness on health behaviours has yet to be explored in people LWBC, but combining strategies that target both elements simultaneously shows promise in this population [101].

Strengths of the study include the use of multiple imputation to overcome any bias or loss of statistical power introduced by only performing complete case analyses [102]. Despite limitations in establishing causality, a strength of the cross-sectional design of this study is that it allowed for the identification of a new area of inquiry that requires attention, and the observed associations can help inform theory development and intervention design [103, 104]. While the ease with which a self-report survey can be distributed to many people is a strength of this type of research, the validity of health research can be threatened by selection biases [105]. In this study, 43% of those sent the initial letter completed the survey. Previous research has demonstrated that people who agree to take part in questionnaire asking about their health behaviours often demonstrate a higher interest in their health and improving health behaviours [106, 107]. Therefore, selection biases may threaten the generalisability of our findings. Limitations of this study also include that this was not an ethnically diverse sample of LWBC, with 90% of participants identifying as white. Another limitation was the use of self-report for recording health behaviours, which might be subject to recall errors and social desirability [108]. Additionally, inherent in research is the limitation of unmeasured confounders that may have contributed to the results. In this study, direct measures of socioeconomic position (e.g., level of income) were not included in the survey and regression model. However, the observed associations remained even after controlling for education level, a variable that has been previously identified as the strongest independent predictor of health behaviours among people LWBC out of three variables indicative of socioeconomic position (including household income and occupation type) [109]. Lastly, some dietary instruments were custom-made for this study, for example, the free sugar measure and this may threaten their validity. However, any adaptations were made based on a review of available measures alongside the main components of the UK diet [47], and modifications were made to validated measures used in previous population level research [54, 110].

## Conclusions

This study reports a similar prevalence of loneliness in people diagnosed with breast, prostate, and colorectal cancer in the UK to that observed in the general population and identifies the need to consider the impact of loneliness on health behaviours in this population. Given the associations between loneliness and physical activity, smoking, and fruit and vegetable consumption, future studies should aim to explore the factors predicting higher levels of loneliness in this population, to identify the people LWBC who are most at risk. Additionally, the mechanisms that might explain the association between loneliness and these health behaviours remain unexplored and future research and care would benefit from exploring why these relationships exist. Particularly following the COVID-19 pandemic and the associated heightened prevalence of loneliness among LWBC [111, 112], future research should aim to take a holistic view of the cancer experience and target aspects of loneliness in health behaviour intervention design.

### Electronic supplementary material

Below is the link to the electronic supplementary material.

Supplementary Material 1

Supplementary Material 2

## Data Availability

The datasets used and/or analysed during the current study are available from the corresponding author upon reasonable request.

## Abbreviations

AICR: American Institute for Cancer Research
AUDIT-C: Alcohol Use Disorders Identification Test consumption questions
BMI: body mass index
CI: confidence interval
DINE FFQ: Dietary Instrument for Nutrition Education Food Frequency Questionnaire
ELSA: English longitudinal Study of Ageing
GCSE: General Certificate of Secondary Education
GLTEQ: Godin leisure time exercise questionnaire
LWBC: living with and beyond cancer
MVPA: moderate-to-vigorous physical activity
NHS: National Health Service
OR: odds ratio
SPSS: Statistical Package for Social Sciences
UK: United Kingdom
WCRF: World Cancer Research Fund
