# Supplementary material for: Prevalence of loneliness and associations with health behaviours and body mass index in 5835 people living with and beyond cancer: a cross-sectional study
Source: BMC Public Health. 2024 Feb 28;24:635. doi: 10.1186/s12889-024-17797-3 (PMC10903019; doi:10.1186/s12889-024-17797-3)
Supplement: Supplementary file 1 — Supplementary Material 1 [file 12889_2024_17797_MOESM1_ESM.docx]

## **Additional file 2**

| **Supplementary Table 2.** Associations between loneliness and health behaviours in people LWBC (completers analysis) | | | | |
| --- | --- | --- | --- | --- |
| **World Cancer Research Fund recommendations**  **(not meeting=0, meeting=1)** | **OR^b^** | **95% CI^c^** | | **p** |
| Moderate-to-vigorous physical activity | 0.88 | 0.78 | 1.03 | 1.05 |
| Fibre | 1.06 | 0.78 | 1.44 | 0.685 |
| Fruit & Vegetables | 0.85 | 0.71 | 1.04 | 0.108 |
| Red Meat | 0.62 | 0.37 | 1.02 | 0.061 |
| Processed Meat | 1.04 | 0.87 | 1.25 | 0.636 |
| Sugar | 0.79 | 0.66 | 0.94 | 0.009* |
| Fat | 0.92 | 0.75 | 1.13 | 0.438 |
| Alcohol | 0.81 | 0.62 | 1.06 | 0.131 |
| Smoking | 0.54 | 0.39 | 0.74 | <0.001* |
| Body mass index | 1.10 | 0.91 | 1.36 | 0.308 |
| ^a^Reference category: lower loneliness (<6 on UCLA loneliness scale). Models adjusted for covariates – age, sex, ethnicity, education, marital status, cancer type, treatments, time since treatment, time since diagnosis, number of comorbidities, cancer spread; ^b^OR=odds ratio; ^c^CI=Confidence interval. *indicates statistical significance at .05 alpha level. | | | | |
