# Supplementary material for: Prevalence of loneliness and associations with health behaviours and body mass index in 5835 people living with and beyond cancer: a cross-sectional study
Source: BMC Public Health. 2024 Feb 28;24:635. doi: 10.1186/s12889-024-17797-3 (PMC10903019; doi:10.1186/s12889-024-17797-3)
Supplement: Supplementary file 2 — Supplementary Material 2 [file 12889_2024_17797_MOESM2_ESM.docx]

## Additional file 1

| **Supplementary Table 1.** Descriptive statistics of completers versus imputed data for loneliness and health behaviours (n=5835) | | |
| --- | --- | --- |
| **Variable** | **Completers (valid %)** | **Imputed data** |
| **Loneliness n(%)** |  |  |
| Higher | 1035 (19.0) | 1229 (21.1) |
| Lower | 4423 (81.0) | 4606 (78.9) |
| Missing | 377 |  |
| **MVPA recommendations n(%)** |  |  |
| Not meeting | 3359 (65.2) | 3838 (65.8) |
| Meeting | 1790 (34.8) | 1997 (34.2) |
| Missing | 686 |  |
| **Fibre recommendations n(%)** |  |  |
| Not meeting | 3914 (85.4) | 4939 (84.6) |
| Meeting | 667 (14.6) | 896 (15.4) |
| Missing | 1254 |  |
| **Fruit and Vegetable recommendations n(%)** |  |  |
| Not meeting | 4006 (70.7) | 4139 (70.9) |
| Meeting | 1659 (29.3) | 1696 (29.1) |
| Missing |  |  |
| **Fat recommendations n(%)** |  |  |
| Not meeting | 1769 (43.5) | 2633 (45.1) |
| Meeting | 2300 (56.5) | 3202 (54.9) |
| Missing | 1766 |  |
| **Sugar recommendations n(%)** |  |  |
| Not meeting | 2663 (49.7) | 2902 (49.7) |
| Meeting | 2694 (50.3) | 2933 (50.3) |
| Missing | 478 |  |
| **Red meat recommendations n(%)** |  |  |
| Not meeting | 139 (2.7) | 186 (3.2) |
| Meeting | 5035 (97.3) | 5649 (96.8) |
| Missing | 661 |  |
| **Processed meat recommendations n(%)** |  |  |
| Not meeting | 2861 (52.0) | 3038 (52.1) |
| Meeting | 2640 (48.0) | 2797 (47.9) |
| Missing | 334 |  |
| **Alcohol recommendations n(%)** |  |  |
| Not meeting | 714 (12.8) | 742 (12.7) |
| Meeting | 4848 (87.2) | 5093 (87.3) |
| Missing | 273 |  |
| **Smoking recommendations n(%)** |  |  |
| Not meeting (i.e., current smoker) | 347 (6.0) | 351 (6.0) |
| Meeting (i.e., current nonsmoker) | 5445 (94.0) | 5484 (94.0) |
| Missing | 43 |  |
| **BMI recommendations n(%)** |  |  |
| Not meeting | 3521 (64.0) | 3747 (64.3) |
| Meeting | 1978 (36.0) | 2088 (35.7) |
| Missing | 336 |  |
| ^a^Meeting and not meeting the recommendations is based on the World Cancer Research Fund recommendations (43). ^b^MVPA=moderate-to-vigorous physical activity. ^c^BMI=body mass index. | | |
